# A Phenomenological Analysis of Knowledge, Impact and Coping Strategies to Climate Change: Experiences of Persons With Albinism

**DOI:** 10.1002/puh2.70291

**Published:** 2026-06-02

**Authors:** Botha Nkosi Nkosi, Segbedzi Cynthia Esinam, Atsu Fortune Selase, Annim Sarah, Ansah Edward Wilson

**Affiliations:** ^1^ Department of Health, Physical Education and Recreation (HPER) University of Cape Coast Cape Coast Ghana; ^2^ E. P. College of Education Amedzofe Ghana

**Keywords:** albinism, carbon footprint, climate change, cutaneous, greenhouse gases, oculocutaneous

## Abstract

**Background:**

Persons with albinism (PWA) in Ghana are disproportionately affected by climate change due to a lack of melanin in their skin, which increases their exposure and vulnerability to the effects of climate change. However, research on this group in Ghana remains limited.

**Purpose:**

To explore the lived experiences of PWA regarding climate change knowledge, impact and adaptation strategies in the Sekondi‐Takoradi Metropolis (STM).

**Methods:**

Data were collected from 17 PWA in the STM, using a five‐item interview guide and analysed through constitutive phenomenological analysis. Recruitment and data collection occurred between 10 June 2021 and 27 August 2023.

**Results:**

We found that PWA in STM possess inadequate knowledge regarding climate change, self‐reported eye and skin conditions and utilize insufficient protection and adaptation strategies.

**Conclusion:**

Although PWA in STM adopt various strategies to cope with climate change effects, these are insufficient for their full protection. The social welfare department should register these individuals to ensure they benefit from government and NGO support. Future research should adopt quantitative approaches and extend this study nationwide.

## Graphical abstract

Persons with albinism in this study have inadequate knowledge regarding climate change, self‐reported eye and skin conditions, and utilize insufficient protection strategies. Although they adopt various strategies to cope with climate change effects, these are insufficient for their full protection. The Social welfare department should register these individuals to ensure they benefit from government and NGO support. Future research should extend this study nationwide using quantitative approaches.

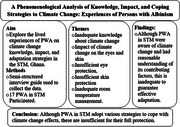

## 1 Introduction

Climate change and associated extreme weather events have become critical global threats to health [1, 2]. However, persons with albinism (PWA) are disproportionately affected by these environmental shifts due to their unique vulnerabilities. Albinism, or achromasia, is a genetic condition characterized by reduced or absent melanin production, primarily affecting the skin, eyes and hair, and is typically accompanied by visual impairment [2, 3]. For instance, oculocutaneous albinism can cause visual impairment due to retinal and iris hypopigmentation, foveal hypoplasia, photophobia, hyperopia, strabismus, nystagmus and loss of stereoscopic perception [4, 5, 6, 7, 8, 9]. Thus, though unexplored in the existing literature, climate change remains a high threat to PWA.

Climate change refers to a significant, long‐term shift in global temperatures and weather patterns [10, 11, 12]. Although some of these changes have occurred naturally throughout history, anthropogenic factors remain the primary drivers. Furthermore, climate change acts as a ‘threat multiplier’ for extreme weather events, such as severe heatwaves, by increasing their frequency, intensity, and unpredictability [1, 2, 13]. The stratospheric ozone layer (SOL) is a naturally occurring gas that filters ultraviolet radiation (UVR) [12, 13, 14], providing an effective screen that protects the Earth from the harmful effects of ultraviolet C (UVC) and a substantial portion of ultraviolet B (UVB) [10, 11], whose effects are detrimental to human health [13, 14]. This function of the SOL is essential for the survival of life on Earth. Anthropogenic greenhouse gas emissions, including carbon dioxide, methane and nitrous oxide from fossil fuel combustion and wildfires, as well as the production of steel, cement, plastics and chlorofluorocarbons (CFCs) used in refrigerants, aerosols and insulation, also remain the primary drivers of climate change and extreme weather events [10, 11], which are exacerbating poor human health [13, 14].

The intersection of climate change and associated extreme weather, like extreme heat, and albinism represents a critical, often overlooked human rights and health crisis [15, 16, 17]. The health and well‐being of PWA is, for example, severely compromised by increased UVR [18, 19]. Unfortunately, PWA already face a multifaceted burden of psycho‐physiological and socio‐economic challenges, including vision impairment, ultraviolet skin damage, poverty, discrimination, social isolation, unemployment and stigmatization [3, 4, 5, 6, 7, 11]. Thus, climate change and extreme weather events exacerbate the psycho‐physiological and socio‐economic challenges faced by this vulnerable population (PWA), particularly in resource‐limited regions [7, 8, 9, 10]. Furthermore, UVR is a primary carcinogen and the leading cause of cutaneous malignancy, which remains one of the most common and serious dermatological conditions among PWA globally [3, 4, 11, 13, 17].

Various factors account for the neglect and limited research about PWA. For instance, in Africa, including Ghana, the prevalence of albinism is estimated at 1 in 5000 [17]. The condition has fostered various myths and misconceptions across the continent. In many regions, derogatory labels are used to stigmatize PWA [2, 6, 7, 8, 9, 18, 19]; including in Tanzania, terms include ‘zeruzeru’ (ghost), ‘mzungu’ (white person), and ‘dili’ (referring to the illicit trade of body parts), whereas ‘agueyoyo’ (white person of the coast) is used in Benin [8, 18, 19]. In Ghana, the Ashanti refer to them as ‘ofri’ or ‘ofridjato’ (scorched or marked person); the Ewe use ‘gesoshi’ (supernatural or incomplete human being) or ‘agbelimoryevu’ (white person); and the Dagati use ‘gbangu’ (supernatural) [7, 8, 9]. It is, therefore, important that the health and well‐being of PWA is considered, especially according to global health agendas. For example, the Sustainable Development Goal (SDG) 3 highlights the connection between climate change and psychosocial challenges faced by vulnerable persons (PWA); SDG 10 draws our attention to how climate change exacerbates existing vulnerabilities (for PWA); and SDG 13 calls for targeted, localized interventions to reduce the effects of climate change on such vulnerable persons (PWA) [20, 21].

Climate change exacerbates the psycho‐physiological and socio‐economic challenges faced by PWA [12, 13, 14, 15, 16]. Higher temperatures, erratic rainfall, severe droughts, land degradation, and water scarcity are reducing incomes and living standards of PWA in Ghana [11]. Furthermore, temperature projections for 2010–2050 indicate a gradual warming across all 16 regions in Ghana [11, 12, 13, 15]. Thus, the impact of climate change on the country is expected to be severe, driven by fluctuations in annual temperatures and precipitation [21]. Several studies have examined PWA in Ghana [2, 4, 10, 11, 15, 18, 19, 21]. Daklo and Obadire [4] explored misconceptions about albinism, whereas Benyah [18] examined how myths, religio–cultural beliefs, and superstitions contribute to the social exclusion of PWA. Furthermore, Affram et al. [2] investigated their subjective well‐being, and Benyah [19] analysed the intersection of religious beliefs, cultural values and albinism. To our knowledge, however, no study has explored climate change knowledge and adaptation strategies among PWA. Therefore, this study examines the lived experiences of PWA regarding their climate change knowledge, impact and adaptation strategies in the Sekondi‐Takoradi Metropolis (STM).

## 2 Materials and Methods

This study employed a descriptive phenomenological (DP) design by Morrow et al. [22] to explore the lived experiences of PWA regarding their climate change knowledge, impacts and adaptation strategies. The DP design is a flexible methodology that can be adapted to various study types, offering a rich and detailed account of participants’ experiences [22]. This approach is effective for probing the perspectives of diverse participants, highlighting similarities and differences and generating unanticipated insights.

According to the leader of the PWA group in STM, although 19 are registered members, many other PWAs within the metropolis remain undocumented. Governmental and non‐governmental organizations (NGOs) occasionally provide education and support to registered members, including free medical screenings, National Health Insurance Scheme registration, hospital bill coverage and start‐up capital for small businesses. However, unregistered PWAs often do not benefit from these services. Registered members were a recognized group of PWA registered with the Department of Social Welfare in the Western Region of Ghana [23, 24]. Because unregistered PWAs often face disadvantages in accessing support [15], they became the focus of this research. Data were collected from 10 June 2021 to 27 August 2023.

### 2.1 Study Area

The study was conducted in the Western Region, one of Ghana's 16 administrative regions [24]. Located in southern Ghana, the region is bordered to the west by the Western North Region and Ivory Coast (Comoé District), to the east by the Central Region, and to the south by the Gulf of Guinea [24]. The regional capital, Sekondi‐Takoradi, is a major twin city situated within a coastal and hilly inland area [24].

### 2.2 Sampling and Recruitment

A total of 31 PWA were contacted for this study; 17 consented to participate, whereas 14 declined. Using a snowballing method, community health officers (CHOs) and social welfare workers (SWWs) in the STM assisted us to recruit and interview the initial 11 PWA. These 11 participants also assisted us via snowball sampling [25] to recruit an additional six PWA. Five participants provided written informed consent, and 12 provided verbal consent, which was documented in the study records or field notes. This included the date, time and location of the informed consent process. Each member of the data collection team who obtained informed consent signed and printed their name on the participants’ records, as approved by the ethical review board. To mitigate selection bias associated with snowball sampling and the potential exclusion of PWAs with severe conditions, we also utilized contact details provided by CHOs and SWWs to ensure sample heterogeneity.

Participants were interviewed individually at their homes. Professional translators from the University of Cape Coast's Department of Ghanaian Languages translated the research instrument from English into the participants’ preferred local languages: Ewe and Akan (Fante/Twi). Each interview lasted between 30 and 45 min, and responses were recorded by hand, as participants declined audio recording. Two team members took field notes simultaneously to prevent a situation where we would not capture information provided by the participant. Data collection continued until saturation was reached. To ensure anonymity and confidentiality, responses were coded rather than linked to personal names [22, 25]. The study purpose and protocols were explained to the participants in Ewe and Akan (Fante/Twi) to ensure they understood the study before taking part. Participants were informed that they could withdraw from the study at any time without penalty. A psychologist was employed to assist with any psycho‐emotional issues during data collection.

### 2.3 Inclusion Criteria

Participants must be individuals with albinism residing in STM, aged 18 years or older, who are available and willing to participate in the study.

### 2.4 Exclusion Criteria

PWA who were unavailable during data collection, those who refused to provide informed consent, and those experiencing emotional distress or poor health were excluded from the study.

### 2.5 Instrument and Ethical Considerations

Data were collected using a 5‐item interview guide (containing 18 prompts) adapted from similar studies [2, 11, 18, 19, 21]. The guide explored the background information of the PWA, including age, sex, education and occupation, as well as their awareness, knowledge and perceptions of the impacts of climate change, along with their coping and adaptation strategies. Sample items included: ‘What do you know about climate change?’ ‘Tell me what you know about climate change’, ‘In what ways have humans contributed to climate change?’ ‘How has climate change or extreme heat affected you?’, ‘How do you protect yourself from the sun and heat?’ and ‘What do you do to cope with extreme heat?’

The draft instrument underwent a rigorous evaluation by the regional chairman of the Ghana Federation of Disability Organizations, four officers from the Department of Social Welfare, and a senior lecturer in special education. The Institutional Review Board of the University of Cape Coast (UCC) approved the study (ID: UCCIRB/EXT/2021/06).

### 2.6 Pre‐Testing

The instrument was pre‐tested with four individuals with physical disabilities at the STM. Additionally, inter‐rater reliability was assessed by four officers from the Department of Social Welfare and a senior lecturer from the Department of Special Education at UCC, Ghana [22, 26]. Subsequently, audio recordings were transcribed and reconciled with field notes to create a complete transcript, which was analysed using the DP [22, 26]. All inconsistencies and grammatical errors identified during pre‐testing were resolved and incorporated into the final instrument to enhance its credibility for data collection.

### 2.7 Data Analysis

Data analysis began after data collection, utilizing a four‐step DP approach [22, 26]. In the first step, two authors (N.N.B. and F.S.A.) read the field notes to understand the participants’ perspectives, identify core phrases and rephrase them in broader terms. This process facilitated the formulation of meanings from the transcripts [22, 26]. In step two, the transcripts were reviewed to identify keywords and phrases, which were then developed into codes. These codes were evaluated for coherence and relevance to the research questions and study purpose by three authors (E.W.A., C.E.S. and N.N.B.). In step three, similar codes were synthesized into themes and screened for relevance. These themes were then refined and organised to ensure alignment with the study's objectives. Three authors (N.N.B., E.W.A. and C.E.S.) resolved thematic discrepancies through extensive reading and discussion [22, 26].

To ensure confidentiality, participant names were anonymized, and ages were redacted (see Table 1). Trustworthiness was established by adhering to the criteria of authenticity, credibility, confirmability, dependability and transferability [22, 26]. Credibility was further enhanced through two follow‐up visits, which allowed participants to verify the accuracy of their views and the subsequent interpretations. On the basis of feedback from these visits, necessary adjustments were made during the fourth step of data analysis. Finally, a report on the PWA's experiences was developed and discussed by all five authors (N.N.B., E.W.A., C.E.S., F.S.A. and S.A.). Consistent with confirmability, every stage of data analysis involved detailed discussion and note‐taking. Participants’ views and experiences were reported accurately and thoroughly to ensure data authenticity [22, 26].

**TABLE 1:** Demographic characteristics of participants.

Variable		Frequency
**Gender**	Male Female	10 7
**Age**	18–28 29–39 40–50	5 9 3
**Level of formal education**	No formal education Primary school JHS/Vocational school SHS	3 4 5 5

Abbreviations: JHS, junior high school; SHS, senior high school.

## 3 Results

Three themes and five sub‐themes emerged from the data. Theme 1 addressed inadequate knowledge regarding climate change. Theme 2 focused on the impact of climate change on PWA, comprising two sub‐themes: impact on the eyes and impact on the skin. Theme 3 concerned inadequate adaptation strategies, consisting of three sub‐themes: insufficient eye protection, insufficient skin protection and room temperature management.

### 3.1 Team 1: Inadequate Knowledge of Climate Change

We found that although PWAs in STM are aware of climate change, their knowledge remains inadequate for developing effective adaptation strategies. Local radio stations serve as their primary source of information. For instance, a male PWA, PMVJ, remarked:

I only hear about climate change in Ghana when the rains fail and drought occurs; the subject is forgotten as soon as the rains are over. In my view, public education on climate change should be handled by health professionals rather than journalists, and should be done from community to community to ensure adequate public awareness.

Meanwhile, some PWAs perceive climate change as an act of God. A female PWA, PFJJ, explained:

 . . . it is true that the weather is becoming increasingly hot. However, what can we do? In my view, this signals the end of the world. Humans do not determine when it rains; no one can control the weather. I believe God is punishing us for our sins. Do you (researcher) agree?

This perspective is concerning, as it may undermine the climate change adaptation and resilience strategies necessary to protect health and that of PWAs.

Another male respondent, PMSSA, stated:

I learned about climate change in school. We were taught that ozone layer depletion is responsible for the current extreme heat. We are experiencing longer periods of drought than in the past. Our teacher advised against deforestation and the burning of rubber and other materials. It is true that ‘when the last tree dies, the last man will die too’.

Although some participants were aware that industrial and anthropogenic activities, such as deforestation, contribute to climate change, they could not specifically explain these connections. For instance, participant, PFSJ, attributed the phenomenon to industrial operations in developed countries, with these remarks:

 . . . I am told that industries contribute to the problem because of the gases they emit into the atmosphere. However, I believe the issue primarily concerns major industries in developed nations, such as the United States, China, France, and the United Kingdom.

### 3.2 Team 2: Impact of Climate Change on PWA

This theme yielded two sub‐themes: impact on the eyes and impact on the skin.

#### 3.2.1 Impact on the Eyes

Participants reported various eye conditions attributed to sun exposure and extreme heat, including eye irritation, redness, swelling, pain, rashes and blurred vision. A participant, PMJJ, remarked:

My eyes swell whenever I walk or work in the sun. In fact, I dropped out of school because of this. I usually experience pain and rashes around my eyes after scratching my eyes.

Another, PFSJ, explained:

Our biggest enemy as PWA is the sun . . . . I truly have passion for football but the sun became my main barrier, as I could not play in the sun for long. However, on days that I played in the sun for longer periods out of “stubbornness”, my eyes ach with sharp sensations which feel like needle pricks. Even in class, my eyes itched, especially on days the classroom became very warm. On such days, my vision becomes distorted and I could neither read nor write.

Participant, PFJT, also explained:

 . . . the discomfort that comes with the heat from the sun is unbearable. I sweat all over my body and my eyelids itch. This becomes worse especially during the dry season.

#### 3.2.2 Impact on the Skin

The skin was identified as one of the most affected body parts. Participants reported various conditions, including irritation, burns, blisters, inflammation and redness. Other affected areas included the lips, nose and eyelids. A participant, PMJJ, observed:

I was advised to wear trousers and long sleeves whenever I went out of the house. However, this comes with so much discomfort as it makes me sweat more than usual . . . I do not get to enjoy the fresh air around. This makes me scratch my skin as the heat builds up, leading to blisters all over my body. In fact, we (PWA) need help and protection from this hash weather. . . . we can neither sleep in the day nor at night.

Another participant, PFSJ, explained:

 . . . so what exactly do you want me to say? . . . because you can see the evidence all over my body. Ok! It is just not a pleasant experience dealing with the sun at all. My mother sells in the market and I sometimes assist her in carrying the items, which exposes me to the burning sun, the result is in itches all over my body.

Some participants struggled to speak during the interview due to the pain from blisters around their mouths.

### 3.3 Team 3: Adaptation Strategies

This theme yielded three sub‐themes: eye protection, skin protection and room temperature management.

#### 3.3.1 Eye Protection Strategies

We found that the eye protection measures adopted by participants were insufficient to guarantee adequate sun protection. These measures included wearing sunglasses, hats and using umbrellas. For instance, a participant, PMVJ, explained:

I normally wear “sunglass”, hat, and occasionally use the umbrella. Though that does not provide me full protection, I still feel somehow assured of my protection from the sun. However, I do not go out at all whenever the sun heats‐up.

Another participant, PMSJ27, explained:

 . . . I have “sunshades” too, but I do not use it because I feel heat around my eyes. The hat also generates heat, but it is better than the “sunshades”.

Although the participants mentioned and displayed their sunshades and hats, it was noted that these were not ultraviolet protection factor (UPF)‐rated; rather, they were ordinary spectacles providing little to no protection.

#### 3.3.2 Skin Protection

We found that PWA in this study employed various, yet inadequate, sun protection strategies on their skin. These included wearing long‐sleeved shirts and trousers or using umbrellas, none of which provided the recommended UPF. Furthermore, PWA experienced social discrimination due to their condition. A participant, PFJJ, noted:

I could not tolerate the discomfort of wearing long sleeves, trousers, and sunshades; instead, I find using an umbrella much more comfortable. Due to the sun's intensity, I prefer staying home to avoid the public's stares. I have often broken down in tears when looking at my face in the mirror.

Additionally, PWA in STM could not afford recommended skin lotions due to their high cost. A participant, PFJJ, remarked:

A teacher once mentioned sunscreen lotion to me, but I could not buy it because it was very expensive… there was no way my parents could afford that.

Furthermore, some participants used frequent bathing as a coping strategy. A participant, MSSA, explained:

…one way I deal with the heat is to bathe regularly, especially after returning from town or school. I bath about four times a day, provided there is enough water.

#### 3.3.3 Management of Room Temperature

We found that PWA adopt various strategies to manage room temperature, including opening windows for ventilation and using ceiling or standing fans. One participant, PMJJ, remarked:

I usually open the windows for fresh air and sleep on a wet towel on days when the room becomes too warm…. although there is a ceiling fan in our room, it is seldom used due to high electricity bills.

Another participant, PFSJ, noted:

 . . . although my mother complains about the electricity bills, I cannot sleep without the ceiling fan. I would prefer an air conditioner, but there was no money to buy one.

## 4 Discussion

Although PWAs in STM were aware of climate change and had some knowledge of its contributing factors, this understanding was insufficient to support effective adaptation. For example, although CFCs are primary drivers of increased UV exposure due to ozone depletion [10, 11, 12, 13, 14], most participants attributed these changes to bushfires, the burning of tires and spiritual factors. The reliance on spiritual explanations reflects a misconception that may increase vulnerability and undermine effective adaptation and coping strategies for PWA. This aligns with a previous study [27], reporting poor climate change knowledge among PWA. Findings of the current study are expected, given the participants’ educational backgrounds.

Previous studies indicate that when PWA are exposed to direct sunlight, extreme heat and intense light, their eyes can be severely affected, resulting in pain and potential vision impairment [18, 19, 20, 21]. Affirming this, PWA in STM reported various eye conditions caused by exposure to sunlight and extreme heat events linked to climate change. Although these experiences may not be unique to PWA in Ghana, the findings align with earlier research [20, 28, 29] suggesting that PWA in Africa are disproportionately impacted by climate change events. Consistent with previous research [19, 20], some participants in this study dropped out of school due to the effects of the sun on their eyes. These experiences have far‐reaching implications for these PWA in rural developing countries, including stigma, isolation and other psychosocial threats.

Furthermore, the nexus between climate change and skin diseases is well‐documented [30, 31, 32, 33, 34, 35]. Ireland [36] identified the lips, nose and periocular regions as high‐risk areas for skin cancer among PWA. Similarly, this study found that non‐unionized PWA in STM suffer from various self‐reported skin conditions due to prolonged exposure to solar radiation and extreme heat, exacerbated by a lack of support compared to their unionized counterparts. Additionally, these PWA often come from disadvantaged backgrounds with limited access to basic social amenities, including healthcare.

Consistent with a previous study [20], we also found that PWA in the STM used non‐prescription spectacles purchased from the open market, which offered minimal protection. Because these ‘sunshades’ lacked ultraviolet protection, their use left PWA vulnerable, as they mistakenly believed these devices could adequately protect their eyes. Previous studies [33, 34, 35, 36] indicate that UPF‐rated sunshades provide effective sun protection. However, consistent with earlier research [28] on PWA in Africa, the current study revealed that these individuals rather face a dual challenge of limited availability and high costs of UPF‐rated sunshades.

Previous studies [36, 37] indicate that sunscreen lotions provide effective skin protection for PWA. However, PWA, particularly in developing countries, often lack access to sunscreen and UV‐protective clothing due to high costs and scarcity [37]. Similarly, this study confirms that PWA in STM often wear clothing that offers inadequate sun protection.

Contrary to previous studies [20, 36], which reported that air conditioners, heat extractors and fans significantly regulate ambient temperatures and provide comfort for PWA, the current study reveals that PWA in the STM cannot afford these options. Common practices, such as frequently washing the face with cold water, sleeping on wet towels and bathing before bed, may offer some relief, but they remain inadequate protection practices against extreme heat.

## 5 Limitations

Although this study offers valuable insight into the experiences of PWA in STM regarding climate change, it has some limitations. The transferability of the findings is limited by the small sample size of 17 participants, the reliance on handwritten notes instead of audio recordings, the geographic delimitation to a single metropolitan area, and the potential for recall, reporting and selection biases. Additionally, possible language translation errors and the intense emotional distress expressed by two participants during the interviews may have influenced the narratives. Fortunately, a clinical psychologist was involved to address these incidents. Despite the study's methodological rigour, we acknowledge that incorporating a quantitative approach could have provided further insights.

## 6 Contributions to Knowledge and Policy

This study offers a nuanced understanding of the vulnerabilities of PWA in STM, addressing a significant research gap while directly advancing SDGs 3, 10 and 13. The qualitative design shifts the narrative beyond statistics to highlight lived experiences, emotional toll, adaptation and coping strategies, and the structural barriers PWA confront regarding how climate change events affect their daily lives and health. The findings directly support SDG 3 by highlighting the psychosocial challenges associated with various eye and skin conditions; SDG 10 by illustrating how climate change exacerbates existing vulnerabilities for PWA in STM, including limited access to sun‐protection resources; and SDG 13 by recommending targeted, localized interventions for PWA in STM. Therefore, it is essential to incorporate measures into climate change policies to protect highly vulnerable populations, including PWA.

## 7 Conclusion

Although PWA in STM were aware of climate change and had reasonable understanding of its contributing factors, this is inadequate to guarantee effective adaptation. For example, spiritual beliefs may foster misconceptions that increase vulnerability and undermine effective adaptation and coping strategies. Participants reported various eye and skin conditions, which caused some to drop out of school and remain housebound. Due to the unavailability and high cost of UPF‐rated materials, as well as the cost of electricity, PWA in STM use ordinary fabrics that provide limited protection against the sun and heat, and they are unable to use fans or air conditioners. Furthermore, the high cost of electricity has forced PWA in STM to adopt alternative, inadequate strategies for managing ambient heat.

Consistent with these findings, we offer recommendations to alleviate the challenges faced by PWA in the STM. The Social Welfare Department, in collaboration with the Ghana Health Service, should develop specialized health education programmes to improve PWA's understanding of albinism, climate change and effective coping strategies. Furthermore, the Social Welfare Department, alongside civil society organizations and NGOs, should provide PWA with sun‐protection resources and necessary assistive devices. Given that high electricity bills are a concern for this group, the government of Ghana should consider subsidizing electricity and water for these vulnerable individuals.

Future research should adopt quantitative approaches to investigate the knowledge of PWA across Ghana regarding climate change and its impact on their psychosocial health.

## Author Contributions

Ansah Edward Wilson, Atsu Fortune Selase and Botha Nkosi Nkosi conceived and designed the study protocols. Segbedzi Cynthia Esinam, Botha Nkosi Nkosi and Annim Sarah conducted data collection and acquisition. Ansah Edward Wilson and Atsu Fortune Selase conducted data management and analyses. Botha Nkosi Nkosi and Annim Sarah wrote the initial manuscript. Segbedzi Cynthia Esinam, Ansah Edward Wilson and Atsu Fortune Selase edited and substantially revised the manuscript. All authors revised and proofread the manuscript for intellectual content and gave consent for the publication of the final version.

## Funding

The authors have nothing to report.

## Ethics Statement

The Institutional Review Board (IRB), University of Cape Coast, Ghana, authorised the study (ID: UCCIRB/EXT/2021/06). We also sought permission from the Sekondi‐Takoradi metropolitan directorate for the study.

## Consent

Each participant also signed or gave verbal informed consent to participate in the study. All authors consented to publish this article.

## Conflicts of Interest

The authors declare no conflicts of interest.

## Data Availability

All relevant data involved in this study are fully reported in the analysis.

## References

[1] B. Astle , M. Buycoa , I. Erob , and S. Reimer‐Kirkhama , “Global Impact of Climate Change on Persons With Albinism: A Human Rights Issue,” Journal of Climate Change and Health 9 (2023): 100190, 10.1016/j.joclim.2022.10019. 36439401 PMC9677557

[2] A. A. Affram , E. Teye‐Kwadjo , and A. A. Gyasi‐Gyamerah , “Influence of Social Stigma on Subjective Well‐Being of Persons With Albinism in Ghana,” Journal of Community & Applied Social Psychology 29, no. 4 (2019): 323–335, 10.1002/casp.2403.

[3] G. Brocco , “Albinism, Stigma, Subjectivity, and Global‐Local Discourses in Tanzania,” Anthropology & Medicine 23, no. 3 (2016): 229–243, 10.1080/13648470.2016.1184009. 27354179 PMC5351792

[4] A. K. Daklo and O. S. Obadire , “Exploring the Experiences of Persons Living With Albinism in Ghana,” Cogent Education 11, no. 1 (2024), 10.1080/2331186X.2024.2335792.

[5] Fondation Pierre Fabre , Preventing Cancers Due to Albinism—Mali (Fondation Pierre Fabre, 2015), https://www.fondationpierrefabre.org/en/our‐programmes/tropical‐dermatology/prevention‐cancers‐albinism/.

[6] S. H. Kiluwa , S. Yohani , and S. Likindikoki , “Accumulated Social Vulnerability and Experiences of Psycho‐Trauma Among Women Living With Albinism in Tanzania,” Disability & Society 39, no. 2 (2022): 400–421, 10.1080/09687599.2022.2072706.

[7] L. N. Dapi , B. A. Tambe , and F. Monebenimp , “Myths Surrounding Albinism and Struggles of Persons With Albinism to Achieve Human Rights in Yaounde, Cameroon,” Journal of Human Rights and Social Work 3, no. 1 (2018): 11–16, 10.1007/s41134-018-0048-5.

[8] A. O. Eballé , C. E. Mvogo , C. Noche , M. E. A. Zoua , and A. V. Dohvoma , “Refractive Errors in Cameroonians Diagnosed With Complete Oculocutaneous Albinism,” Clinical Ophthalmology (Auckland NZ) 7, no. 1 (2013): 1491–1495, https://dio.org/10.2147/OPTH.S38194. 10.2147/OPTH.S38194 PMC3726607 23901257

[9] A. Olagunju , M. Mabhala , G. Buck , and L. Taylor , “Being Different: What It Means to be a Person With Albinism in Nigeria,” Disability & Society 40 (2024): 1872–1896, 10.1080/09687599.2024.2380502.

[10] G. Buta , Ghana Climate Change Factsheet 2024 (United States Agency for International Development (USAID), 2024), https://www.usaid.gov/sites/default/files/2024‐08/International%20Youth%20Day%20240809.pdf.

[11] World Bank Group , Ghana Country Climate and Development Report. CCDR Series (World Bank Group, 2020), https://hdl.handle.net/10986/38209.

[12] G. Boateng , Saving Our Planet: Regional Collaboration on Effective Climate Change Actions May Provide Answers (African Center for Economic Transformation, 2019), https://acetforafrica.org/research‐and‐analysis/insights‐ideas/commentary/saving‐our‐planetregional‐collaboration‐on‐effective‐climate‐change‐actions‐may‐provide‐answers/.

[13] D. Estrin , The Connection Between Climate Change and Human Rights (Centre for International Governance Innovation (CIGI), 2016), https://www.cigionline.org/multimedia/connection‐between‐climate‐change‐and‐humanrights/.

[14] Standing Voice , Manual of Best Practice: Ground Breaking Guide to Skin Cancer Prevention (Standing Voice, 2019).

[15] United Nations Environment Programme (UNEP) , COVID‐19 is a Wake‐Up Call: Ghana to Develop National Plan for Climate Adaptation (UNEP, 2020), https://www.unep.org/news‐and‐stories/pressrelease/covid‐19‐wake‐call‐ghana‐develop‐national‐plan‐climate‐adaptation.

[16] World Bank , Under the Same Sun: The Struggle for Social Inclusion of People With Albinism (World Bank, 2015), https://www.worldbank.org/en/news/feature/2015/08/05/under‐the‐same‐sun‐thestruggle‐for‐social‐inclusion‐of‐people‐with‐albinism.

[17] R. Phatoli , N. Bila , and E. Ross , “being Black in a White Skin: Beliefs and Stereotypes Around Albinism at a South African University,” African Journal of Disability 4, no. 1 (2015): 106–122, https://dio.org/10.4102/ajod.vd4i.106. 28730019 10.4102/ajod.v4i1.106 PMC5433467

[18] F. E. K. Benyah , “Albinism and Social Exclusion in Ghana,” in Contemporary Discourse in Social Exclusion, Are Albinos People Like Us?, ed. A. A. Alemanji , C. M. Meijer , M. Kwazema , and F. E. K. Benyah (Springer International Publishing, 2023), 143–170, 10.1007/978-3-031-18180-1_7.

[19] F. E. K. Benyah , “Equally Able, Differently Looking: Discrimination and Physical Violence Against Persons With Albinism in Ghana,” Journal for the Study of Religion 30, no. 1 (2017): 161–188, https://dio.org/10.17159/2413‐3027/2017/v30n1a7.

[20] B. M. Mabuya , “The Three Little Houses: A Comparative Study of Indoor and Ambient Temperatures in Three Low‐Cost Housing Types in Gauteng and Mpumalanga, South Africa,” International Journal of Environmental Research and Public Health 17, no. 10 (2020): 3524, 10.3390/ijerph17103524. 32443548 PMC7277949

[21] C. Cameron , Climate Change Financing and Aid Effectiveness: Ghana Case Study (UNEP, CTCN, 2011), http://www.eldis.org/document/A61721.

[22] R. Morrow , A. Rodriguez , and N. King , “Colaizzi's Descriptive Phenomenological Method,” Psychologist 28, no. 8 (2015): 643–644, https://eprints.hud.ac.uk/id/eprint/26984/.

[23] Western Regional Coordinating Council (WRCC) , Profile of Region (WRCC, 2022), https://wrcc.gov.gh/regional‐profile/.

[24] Ghana Statistical Services , 2021 Population and Housing Census (Ghana Statistical Services, 2021), https://census2021.statsghana.gov.gh/.

[25] M. Wendy , Edmonds Introduces and Defines Sampling. Using Snowball Sampling to Recruit Participants (Sage Publications Ltd, (2023), 10.4135/9781529667547.n1.

[26] M. M. Hennink , B. N. Kaiser , and V. C. Marconi , “Code Saturation Versus Meaning Saturation: How Many Interviews Are Enough?,” Qualitative Health Research 27 (2017): 591–608, 10.1177/1049732316665344. 27670770 PMC9359070

[27] World Bank , Ghana—Climate Change Knowledge Portal Summary (World Bank, 2021), https://climateknowledgeportal.worldbank.org/country/ghana.

[28] C. Scheerens , “Tackling Adverse Health Effects of Climate Change and Migration Through Intersectoral Capacity Building in Sub‐Saharan Africa,” BJGP Open 4, no. 2 (2020): bjgpopen20X101065, 10.3399/bjgpopen20x101065. PMC7330223 32295792

[29] Advantage Africa , Improving the Lives of People With Albinism (Advantage Africa, 2021), https://www.advantageafrica.org/life‐opportunities‐for‐children‐with‐albinism.

[30] J. K. Ganle , E. Otupiri , B. Obeng , A. K. Edusie , and A. Ankomah , and R. Adanu , “Challenges Women With Disability Face in Accessing and Using Maternal Healthcare Services in Ghana: A Qualitative Study,” PLoS ONE 11, no. 6 (2016): e0158361, 10.1371/journal.pone.0158361. 27347880 PMC4922658

[31] E. Batha , Musician With Albinism Says Coronavirus Raises Risk of Attack (Reuters, 2020), https://www.reuters.com/article/us‐health‐coronavirus‐zambia‐albinism‐tr‐idUSKCN25F0Z0.

[32] M. H. Brilliant , “Albinism in Africa: A Medical and Social Emergency,” International Health 7 (2015): 223–225, 10.1093/inthealth/ihv039. 26063702

[33] C. Y. Wright , Africa Needs a Health Policy to Help People With Albinism (Conversation, 2015), https://theconversation.com/africa‐needs‐a‐health‐policy‐to‐help‐people‐with‐albinism‐43870.

[34] F. S. Nuvey , “Poor Mental Health of Livestock Farmers in Africa: A Mixed Methods Case Study From Ghana,” BMC Public Health [Electronic Resource] 20, no. 1 (2020): 825, 10.1186/s12889-020-08949-2. 32487132 PMC7268426

[35] C. Y. Wright , “Socio‐Economic, Infrastructural and Health‐Related Risk Factors Associated With Adverse Heat Health Effects Reportedly Experienced During Hot Weather in South Africa,” Pan African Medical Journal 34 (2019): 40–40, 10.11604/pamj.2019.34.40.17569. 31762907 PMC6859010

[36] E. Ireland , Care of *People With Albinism During* the *Time* of COVID*‐19* (Esther Ireland, 2021): 23, https://www.esther.ie/care‐of‐people‐with‐albinism‐during‐the‐time‐of‐covid‐19/.

[37] N. Naicker , “Indoor Temperatures in Low Cost Housing in Johannesburg, South Africa,” International Journal of Environmental Research and Public Health 14, no. 11 (2017): 1410, 10.3390/ijerph14111410. 29156558 PMC5708049

